# Engaging patients in antimicrobial stewardship: co-designed educational tool to improve periprocedural care through de-implementation of guideline-discordant antimicrobial use

**DOI:** 10.1017/ash.2023.420

**Published:** 2023-09-28

**Authors:** Hawra Al Lawati, Marlena Shin, Rebecca Lamkin, Tyler Thompson, Isabella Epshtein, Hillary Mull, Dipandita Basnet Thapa, Dimitri Drekonja, Maria C. Rodriguez-Barradas, Teena Huan Xu, Howard Gold, A. Rani Elwy, Judy Strymish, Westyn Branch-Elliman

**Affiliations:** 1 Beth Israel Deaconess Medical Center, Boston, MA, USA; 2 VA Boston Healthcare System Center for Healthcare Organization and Implementation Research (CHOIR), West Roxbury, MA 02132, USA; 3 Independent researcher; 4 VA Boston Healthcare System, Department of Medicine, Section of Infectious Diseases, West Roxbury, MA, USA; 5 Boston University Chobanian & Avedisian School of Medicine, Boston, USA; 6 Veterans Affairs Minneapolis Health Care System, Minneapolis, MN, USA; 7 University of Minnesota Medical School, Minneapolis, MN, USA; 8 Michael E. DeBakey Veterans Affairs Medical Center, Houston, TX, USA; 9 Baylor College of Medicine, Houston, TX, USA; 10 Brown University School of Medicine, Providence, RI, USA; 11 Harvard Medical School, Boston, MA, USA

## Abstract

Effective de-implementation models often include replacement of an ineffective practice with an alternative. We co-developed patient education materials as a replacement strategy for inappropriate post-procedural antibiotics in cardiac device procedures. Lessons learned and developed materials may be used to promote infection prevention in other periprocedural settings.

## Background

Effective antimicrobial stewardship interventions, particularly those that target the de-implementation of inappropriate antimicrobial prescribing, are a national priority. Traditionally, antimicrobial stewardship interventions focus on prescribing providers. However, recent data suggest that antimicrobial overuse can be driven by non-physician providers, patients, and process factors.^1^ Stewardship interventions directed only toward prescribers without addressing these other drivers of antimicrobial use are thus unlikely to be effective.

Multi-level interventions that target and engage various members of the healthcare system (providers, nurses, and patients) to optimize medication use are more successful than those that only target prescribers.^2^ One study demonstrated that providing patients with their medication list prior to their appointment and encouraging them to discuss the ongoing need for types of medications (eg, proton pump inhibitors) was an effective patient-initiated intervention that reduced unnecessary medication use.^3^ However, this work focused on medications prescribed chronically, rather than short-term medications, as is more commonly the case with antimicrobials. The Surgical Care Improvement Project included early antimicrobial discontinuation after skin closure as a core measure, however, was limited in scope to major inpatient surgeries.^4^ Diffusion of these practices to uncovered clinical areas is limited and de-implementation strategies to improve perioperative stewardship are needed.^5^

Prolonged antimicrobial use following cardiovascular implantable electronic device (CIED) interventions is common, occurring following approximately half of all procedures.^6^ While traditional physician-directed stewardship interventions focus on simple removal (eg, stopping antimicrobial use), most effective models of de-implementation include removal and replacement strategies (eg, replacing an effective practice such as prolonged antimicrobial use with another intervention). Thus, the aim of this study was to co-develop patient-facing stewardship education materials on periprocedural antimicrobial use in CIED procedures through qualitative feedback from key stakeholders (patients and antimicrobial stewardship providers). Materials developed were designed to be used as part of a bundle of de-implementation strategies to improve periprocedural prescribing.^7^

## Methods

Development of the patient-facing stewardship materials occurred in several steps. First, study investigators identified key stewardship messages from publicly available resources, such as the Agency for Healthcare Research and Quality. Images and messages were collected and adapted to the setting of CIED procedures. These images and messages were then provided to a graphic medicine specialist who drafted a two-page educational handout for patients.

After initial development, materials were presented to key stakeholders (providers and patients) for qualitative feedback and iteratively revised as part of a co-creation process. Feedback was first elicited from infectious diseases providers who participate in antimicrobial stewardship activities and are participating in a quasi-experimental study to promote de-implementation of guideline-discordant antimicrobial use.^8^ Next, study investigators, along with the graphic medicine specialist, presented the educational materials to a Veteran Engagement in Research Group (VERG) and obtained qualitative feedback from veterans. After incorporating veterans’ feedback, the materials with updated messaging were re-presented to providers to obtain a final round of comments. Recommendations collected through the qualitative feedback process were used to iteratively update, modify, and evolve the educational materials until consensus from providers about the messaging was reached.

## Results

**Figure 1.**
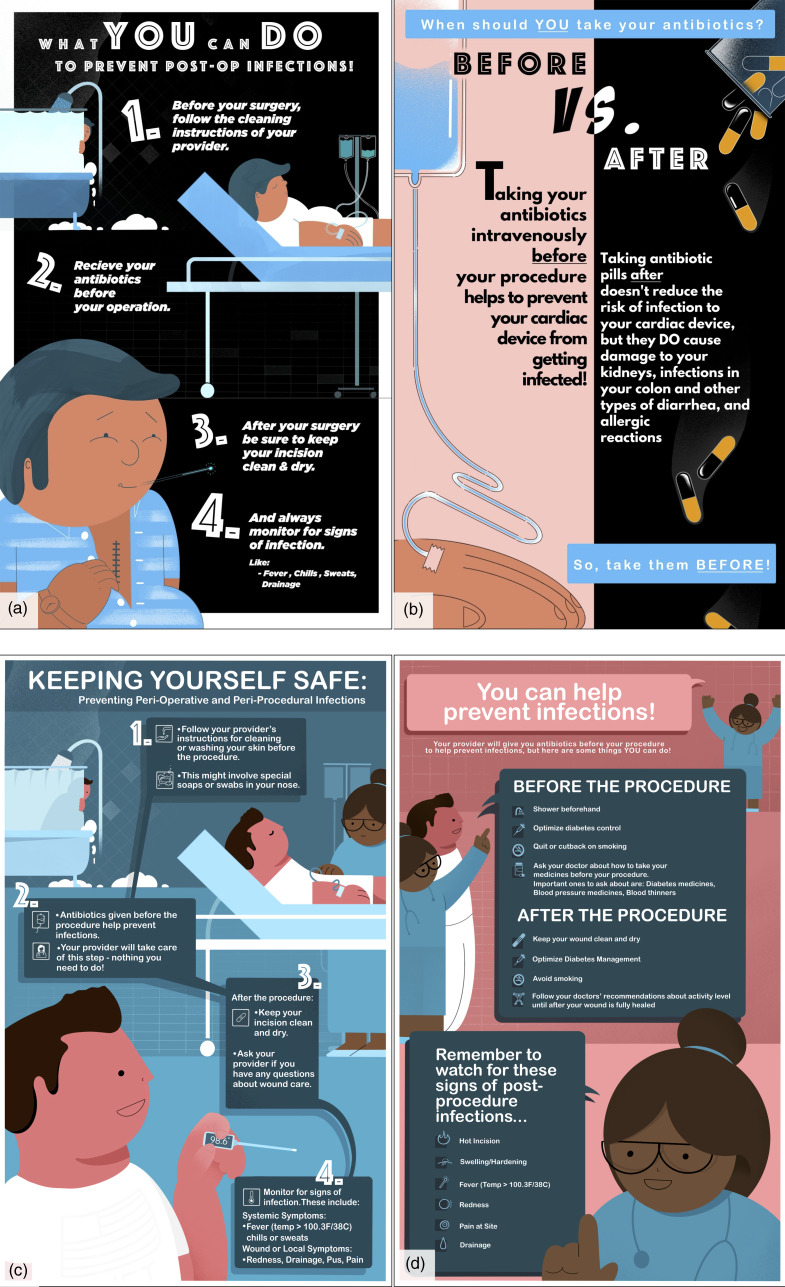
Patient-facing stewardship material. (A) & (B) Initial version; (C) & (D) Final version.

After initial development, five infectious diseases specialists involved in antimicrobial stewardship and four veteran participants reviewed the educational materials and provided feedback. The initial version of the materials contained two major components: a front side, which focused on strategies that patients can complete to prevent periprocedural infections, and a back side, which focused on the ineffectiveness of post-procedural antimicrobials and the potential harms of inappropriate antimicrobial use, such as *C. difficile* infection (Figure 1, Panels A and B).

Both veteran patients and providers expressed a lack of support for messages that encouraged patients to question the utility of prolonged antimicrobial prescribing in the periprocedural setting. Providers also expressed that they did not feel comfortable providing negative messages about harms to patients or encouraging patients to question provider decision-making.

Major changes made to the materials based upon the feedback can be distilled into three main themes: (1) shifting focus from harms to benefits of antibiotics, (2) passive language relating to antibiotic administration, and (3) a focus on infection prevention (Table 1). The final version (Figure 1, Panels C and D) ultimately included an entirely revised back page, which supported positive messaging about interventions that are effective for preventing procedure-related infections, rather than focusing on ineffective and harmful effects of inappropriate antimicrobial use.

**Table 1.** Themes that emerged from feedback and examples of changes made

Theme	Summary of feedback received with sample quotes	Examples of changes made
**Shifting focus from harms to benefits of antibiotics**	Preference to move away from language that emphasizes harms of antibiotics or negative outcomes in general. “*I might not oversell the risk of adverse drug reactions”* and suggested when listing adverse reactions to change from antibiotics *“do cause…” to “can cause…”* - Provider	Phrases that indicated possible harms of antibiotics such as “**they do cause damage to kidneys, diarrhea or allergic reactions**” were replaced by phrases that focused on benefits of antibiotics eg “**one of the best ways to reduce your risk of infection**.”
**Passive language relating to antibiotic administration**	Clinicians and veterans advocated to change the initial wording of “*taking your antibiotics*” as this may not reflect the practical reality that patients often don’t have agency in how or when they receive antibiotics in the procedural setting. *“The patient has no control on this step.”*- Provider *“Patients don’t really have agency to take the IV antibiotics, it is passive, so maybe ‘Getting your antibiotics’ instead of “Taking”?”*- Provider Regarding the title of the first page “what you can do”: *“Adjust language. As a patient, antibiotics is something providers will provide to patients so they, as patients, don’t have control over this.”*- Patient	“**Taking your antibiotics**” and “**receive your antibiotics**” were changed to “**antibiotics given**” or “**your provider will give you antibiotics**.” Changed front page messaging “**what you can do to prevent post-op infections!**” to “**keeping yourself safe: preventing peri-operative and periprocedural infections**”
**Focus on infection prevention**	“*Is patient really in control of antibiotics, vs asking about antibiotics, surgery vs procedure?*”- Provider	Shifting the theme away from antibiotic prescribing based on feedback led to emphasizing infection prevention measures that patients have more agency over. The entire back page became devoted to “**what a patient can do**” to prevent infections.

## Discussion

Reducing inappropriate antimicrobial use can be viewed as a de-implementation challenge. Most implementation science models for de-implementation emphasize removal and replacement strategies to promote practice change.^9^ During our formative work, we found that ongoing antimicrobial use in the cardiac electrophysiology laboratory is a multi-level problem, driven not only by provider practice patterns but also by other clinical care providers and process elements that contribute to the sustainment of the guideline-discordant practice.^1^ Thus, we sought to develop educational materials to address these barriers by giving providers other tools for promoting CIED infection prevention that would also engage patients in their own care. Our initial plan for this implementation strategy was to directly engage patients in antimicrobial stewardship activities, similar to work in de-prescribing of chronic medications demonstrating the effectiveness of patient-driven processes.^3^

Based on traditional stewardship approaches, initial messaging in the materials focused on the harms of antibiotics. However, during the co-creation process, we found that negative messaging about the potential harms of inappropriate antibiotics and messaging related to patients asking providers about the necessity of post-procedural antibiotics were poorly received. The consistent feedback we received across key stakeholders about positive messaging (eg, what patients can do to prevent CIED infection) may suggest stronger support for replacement of processes (eg, focusing on effective interventions) compared to solely removal of processes (eg, focusing on harms of post-procedural antibiotics).^9–11^

Reluctance to encourage questioning post-procedure antibiotic prescriptions was expressed by patients and stewardship providers and in the context of guideline-discordant use.^8^ This patient feedback was in contrast with other work that was found successful in de-prescribing using patient-targeted materials that included an emphasis on drug adverse effects.^3^ However, this successful study was completed in primary care and targeted long-term medications, which may be more conducive to shared decision-making discussions. While feedback in our study was elicited from key stakeholders (physicians and patients), study limitations include that this was from a small number of qualitative discussions rather than a standardized process, such as focus group or formal survey. However, participants in these discussions are the end-users of the products, and thus their buy-in and support are critical for adoption and dissemination of materials. The patients in this study were part of the Veterans Affairs (VA) hospital and perceptions may be different from non-VA populations.

In summary, positive messaging about the benefits of antibiotics and an emphasis on actionable infection prevention strategies were more acceptable to patients and providers than a focus on antibiotic harms in the creation of periprocedural patient-facing stewardship education materials, which can be widely used to expand antimicrobial stewardship practices and messages to clinical care settings with limited coverage, such as the electrophysiology laboratory (final materials available for download in the Supplementary Materials). Future work will focus on assessing the impact of the educational material in a de-implementation trial within real-world clinical settings.^7^

## Supporting information

Al Lawati et al. supplementary material Al Lawati et al. supplementary material

## Supplementary material

For supplementary material accompanying this paper visit http://doi.org/10.1017/ash.2023.420. click here to view supplementary material
