# Supplementary material for: Engaging patients in antimicrobial stewardship: co-designed educational tool to improve periprocedural care through de-implementation of guideline-discordant antimicrobial use
Source: Antimicrob Steward Healthc Epidemiol. 2023 Sep 28;3(1):e163. doi: 10.1017/ash.2023.420 (PMC10644164; doi:10.1017/ash.2023.420)
Supplement: Al Lawati et al. supplementary material [file S2732494X23004205sup001.pdf]

KEEPING YOURSELF SAFE:

Preventing Peri-Operative and Peri-Procedural Infections

1.

- Follow your provider's instructions for cleaning or washing your skin before the procedure.
- This might involve special soaps or swabs in your nose.

2.

- Antibiotics given before the procedure help prevent infections.
- Your provider will take care of this step - nothing you need to do!

3.

After the procedure:

- Keep your incision clean and dry.
- Ask your provider if you have any questions about wound care.

98.6°

4.

Monitor for signs of infection. These include:

Systemic Symptoms:

- Fever (temp > 100.3F/38C) chills or sweats

Wound or Local Symptoms:

- Redness, Drainage, Pus, Pain

You can help prevent infections!

Your provider will give you antibiotics before your procedure to help prevent infections, but here are some things YOU can do!

BEFORE THE PROCEDURE

- Shower beforehand
- Optimize diabetes control
- Quit or cutback on smoking
- Ask your doctor about how to take your medicines before your procedure.
  Important ones to ask about are: Diabetes medicines, Blood pressure medicines, Blood thinners

AFTER THE PROCEDURE

- Keep your wound clean and dry
- Optimize Diabetes Management
- Avoid smoking
- Follow your doctors' recommendations about activity level until after your wound is fully healed

Remember to watch for these signs of post-procedure infections...

- Hot Incision
- Swelling/Hardening
- Fever (Temp > 100.3F/38C)
- Redness
- Pain at Site
- Drainage
